# Synthesis and Characterization of Novel Ruthenium(III) Complexes with Histamine

**DOI:** 10.1155/2010/183097

**Published:** 2010-06-02

**Authors:** Jakob Kljun, Saša Petriček, Dušan Žigon, Rosana Hudej, Damijan Miklavčič, Iztok Turel

**Affiliations:** ^1^Faculty of Chemistry and Chemical Technology, University of Ljubljana, Aškerčeva 5, 1000 Ljubljana, Slovenia; ^2^Department of Environmental Sciences, Jožef Stefan Institute, Jamova c. 39, 1000 Ljubljana, Slovenia; ^3^Faculty of Electrical Engeneering, University of Ljubljana, Tržaška 25, 1000 Ljubljana, Slovenia

## Abstract

Novel ruthenium(III) complexes with histamine [RuCl_4_(dmso-*S*)(histamineH)] · H__2__O (**1a**) and [RuCl_4_(dmso-*S*)(histamineH)] (**1b**) have been prepared and characterized by X-ray structure analysis. Their crystal structures are similar and show a protonated amino group on the side chain of the ligand which is not very common for a simple heterocyclic derivative such as histamine. Biological assays to test the cytotoxicity of the compound **1b** combined with electroporation were performed to determine its potential for future medical applications in cancer treatment.

## 1. Introduction

Before the discovery of cisplatin and its successful use as an anticancer agent in the 70s, metal complexes were rarely considered useful for medical applications. Afterwards, new cisplatin analogues (carboplatin, oxaliplatin) were developed and introduced into clinical use [1–3]. The development of platinum-drug resistance in cancer patients, the general toxicity and severe side effects of platinum drugs however required a different approach in the research of anticancer metal complexes [2].

Complexes of different metals were prepared and tested. Two ruthenium compounds, NAMI-A and KP1019 [4–6], are currently among the most successful candidates to enter the clinical practice. These two ruthenium(III) complexes have an octahedral geometry and contain four in-plane chlorido ligands as well as one dimethylsulfoxido and one imidazolo (NAMI-A) or two indazolo ligands (KP1019) in trans positions. The aforementioned compounds are the chosen representatives of two larger classes of compounds bearing nitrogen-bound aromatic heterocycles developed by the Alessio and Keppler research groups, respectively [4, 7, 8]. 

**Scheme 2:**
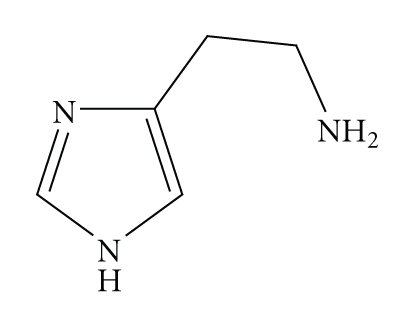
Histamine.

Histamine (4-(2-Aminoethyl)-1*H*-imidazole, see Scheme 2) is a molecule that performs various functions in the body, the most important being the gastric acid secretion and the triggering of the symptoms of an allergic reaction such as vasodilatation, bronchoconstriction, bronchial muscle contraction, pain, and itching.

Most of the metal complexes in use in current cancer treatment have an intracellular target and the plasma membrane can represent a considerable barrier. Electroporation or electropermeabilization is a process where exposing cells to specific electrical pulses results in temporary formation of hydrophilic pores in the cell membrane. Thus temporally increased cell permeability enables extracellular molecules with otherwise hampered transmembrane transport to enter the cells. Electroporation is used in a variety of biotechnological and medical applications. It has been proven that combining electroporation with chemotherapy potentiates the cytotoxicity of drugs when the drugs' efficacy is limited by its uptake in the cell [9–13].

The aim of this study was to prepare and characterize new ruthenium NAMI-type compounds, to test their *in vitro* cytotoxicity and study the influence of electroporation on the cytotoxic activity of the synthesized compounds.

## 2. Experimental

### 2.1. Materials and Instruments

Ruthenium(III) chloride hydrate, histamine dihydrochloride, and the solvents (dimethylsulfoxide, concentrated hydrochloric acid, methanol, ethanol, and acetone) were purchased by Sigma-Aldrich and used without further purification.

#### 2.1.1. Infrared Spectroscopy

Infrared spectra (ATR) were recorded on a Perkin-Elmer Spectrum 100 spectrometer. The measurements were made in the range from 4000 to 600 cm^−1^.

#### 2.1.2. Electrospray Ionization Mass Spectrometry

Mass measurements were run on a hybrid quadrupole time of flight mass spectrometer Q-Tof Premier (Waters Micromass, Manchester, UK), equipped with an orthogonal Z-spray electrospray (ESI) interface.

Water sample solution was introduced directly through syringe pump at a flow rate 5 *μ*L/min. Compressed nitrogen (99.999%, Messer Slovenia) was used as both the drying and the nebulizing gas. The nebulizer gas flow rate was set to approximately 20 L/h and the desolvation gas flow rate to 600 L/h. A cone voltage of 30 V and a capillary voltage of 2.9 kV were used in positive ion mode. The desolvation temperature was set to 150°C and the source temperature to 100°C. The mass resolution of approximately 9500 fwhm was used for determination of elemental composition with TOF mass spectrometer. MS and MS/MS spectra were acquired in centroid mode over an m/z range of 50–1000 in scan time 1 s and inter scan time 0.1 s. The detector potential was set to 1850 V. Reproducible and accurate mass measurements at approximate 10000 mass resolution were obtained using an electrospray dual sprayer with leucine enkephalin ([MH]^+^ = 556.2771) as a reference compound, introduced into the mass spectrometer alternating with a sample solution. 

The data station operating software was Mass Lynx v. 4.1 (Micromass, Manchester).

Interpretation of peaks in mass spectra and identification of particular fragment ions were confirmed with elemental composition mass measurements of these ions at high resolution.

#### 2.1.3. CHN Elemental Analysis

Elemental analyses were performed on a Perkin-Elmer Elemental analyzer 2400 CHN.

#### 2.1.4. X-Ray Structure Analysis

X-ray diffraction data were collected on a Nonius Kappa CCD diffractometer at 150 K for compound **1a** and at room temperature for compound **1b** using graphite monochromated Mo-*K_*α*_* radiation and processed using DENZO [14] program. The structures were solved using SIR92 [15]. A full-matrix least-squares refinement on *F* magnitudes with anisotropic displacement factors for all nonhydrogen atoms using SHELXL [16] was employed. The drawings were prepared with the Mercury program [17]. Hydrogen atoms were placed in geometrically calculated positions and were refined using a riding model.

The crystallographic data for compounds **1a **and** 1b** have been deposited with the CCDC as supplementary material with the deposition numbers CCDC 759890 and 759818, respectively (see Supplementary Material available online at doi:10.1155/2010/183097). 

### 2.2. Syntheses

**Scheme 3:**
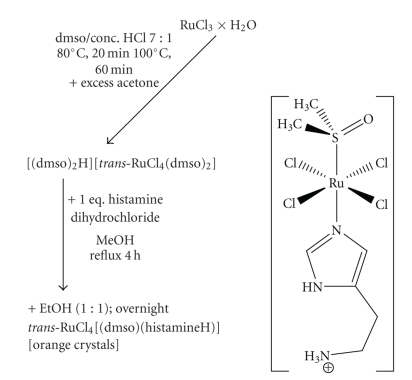
Two step synthesis and structure of the novel ruthenium complex with histamine.

Syntheses of [RuCl_4_(dmso-*S*)(histamineH)]*·*H_2_O **(1a)** and [RuCl_4_(dmso-*S*)(histamineH)] (**1b**): the synthesis of compounds **1a** and **1b** consists of two steps (see Scheme 3). The first step is the preparation of a ruthenium precursor **P1** (dmso_2_H)[*trans*-RuCl_4_(dmso-*S*)_2_]. This compound was prepared according to the literature [18]. 200 mg of **P1** and 70 mg of histamine dihydrochloride were dissolved in 15 mL of methanol and refluxed for 4 hours. After a slow evaporation of the solvent, only a few red crystals of **1a** were obtained. The X-ray structure analysis showed the presence of a solvate water in a molecule. Despite considerable effort, we were not able to obtain such crystals again. Crystals of compound **1b** were later obtained by adding 15 mL of ethanol to the reaction mixture and the solution was left to stand in an open flask. Overnight snowflake-like crystalline orange solid formed. The crystals were washed with cold acetone and diethylether and dried at 50°C for 30 minutes. Similar crystals although of lower quality were obtained by addition of isopropanol, n-pentanol or ethyl acetate, instead of ethanol, to the reaction mixture.

IR (ATR): 3226 (sh), 3115 (s), 2923 (w), 1597 (w), 1578 (w), 1504 (sh), 1480 (m), 1402 (m), 1231 (w), 1111 (sh), 1082 (s), 1016 (s), 968 (s), 934 (s), 928 (sh), 840 (s), 684 (w), 658 (m) cm^−1^.

CHN (only **1b**): Calc. C 19,41%, H 3,72%, N 9,70%; Found: C 19,78%, H 3,67%, N 9,49%.

ESI-MS (in H_2_O solution): m/z 361, 325, 299, 264.

### 2.3. Biological Activity Assays

Murine melanoma cell line B16F1 (European Collection of Cell Cultures, UK) was tested *in vitro* to determine cytotoxic effect of compound **1b** in combination with or without electroporation. B16F1 cell suspension (22·10^6^ cells/mL) was prepared in low conductive electroporation buffer (10 mM (Na_2_HPO_4 _/NaH_2_PO_4_, pH 7.4) with 1 mM MgCl_2 _  and  250 mM sucrose). Different concentrations of compound **1b** were added to cell suspension to reach final concentrations of 0.01, 0.1, and 1 mM. Immediately after incubation (<0.5 min), a drop of cell suspension was placed between two flat parallel stainless-steel electrodes 2 mm apart and a train of electric pulses (8 pulses, 800 V/cm, 100 *μ*s, 1 Hz) was applied with an electroporator Cliniporator (Igea, Carpi, Italy). The same procedure without electric pulses was used for cells exposed to different concentrations of **1b** without electroporation. In addition, we tested cytotoxic effect of **1b** after prolonged incubation time (60 min, without electroporation). Cell viability was measured 72 h after treatment using the MTS-based Cell Titer 96 AQ_ueous_ One Solution Cell Proliferation Assay (Promega, Madison, WI, USA). Absorption at 490 nm wavelength (A_490_) was measured with a spectrophotometer Tecan infinite M200 (Tecan, Switzerland). Cell viability (C.V.) of treated cells (tr) was calculated using the formula: C.V. = (A_490_)_tr _/(A_490_)_c_ × 100[%], taking the cell viability of the control I as 100%. Statistical analysis was performed using One-Way ANOVA test and SigmaStat statistical software (SPSS, Chicago, USA). 

## 3. Results and Discussion

### 3.1. Synthesis and Crystal Structure

Several complexes of the NAMI and KP families bearing nitrogen-bound simple heterocycles or smaller biologically active molecules were already synthesized and investigated for biological applications [4, 7, 19, 20]. Most of the NAMI analogues were synthesized by mixing a suspension of **P1** in acetone and adding the equivalent amount of the ligand and then recrystallizing the product either from hot acetone or other solvents. In our case this synthetic route was not appropriate due to the low solubility of compound **1b** in acetone or other solvents except in water and methanol. The reaction was thus performed in methanol and another less volatile and less polar solvent was added later. Similar crystals although of lower quality were obtained by addition of isopropanol, n-pentanol, or ethyl acetate instead of ethanol to the reaction mixture. The crystals were cut and were suitable for X-ray structure analysis. The experimental data is shown in Table 1.

**Figure 1:**
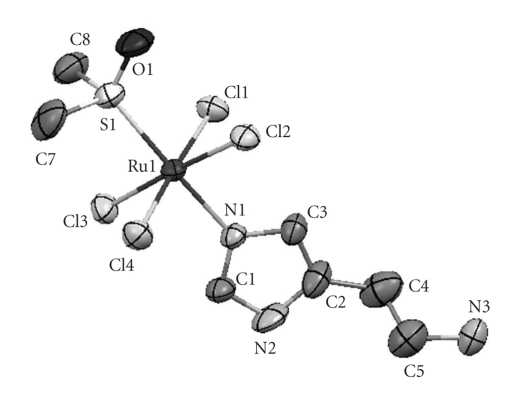
Asymmetric unit of the crystal structure of complex **1b**. The ellipsoids are shown at 50% probability.

The asymmetric unit of compound **1b** is shown in Figure 1. Selected bond lengths and hydrogen bond short contact distances are presented in Tables 2 and 3, respectively.

**Figure 2:**
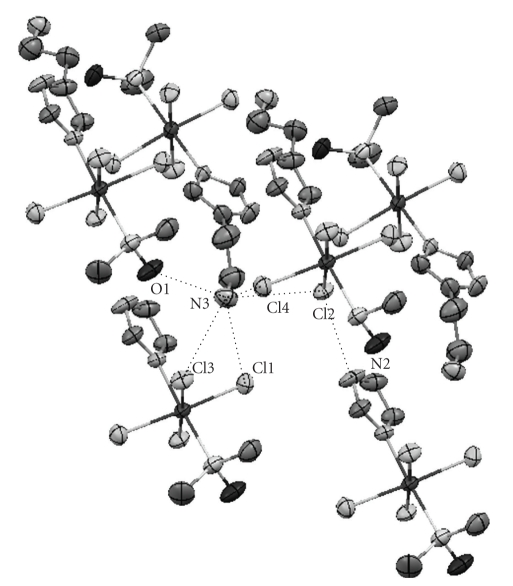
The hydrogen bonds in the crystal structure of compound **1b**. The ammonioethyl groups of the lower two asymmetric units are omitted for clarity.

The crystal structure of compounds **1a **and** 1b** does not differ significantly from the other NAMI-type compounds. Compounds **1a **and** 1b** have a distorted octahedral geometry with four in-plane chloride anions surrounding the Ru(III) ion in addition to a sulfur atom from an S-bonded dimethylsulfoxide molecule and a nitrogen atom from the histamine molecule in axial positions. The main difference to most of the NAMI-type compounds which are anionic complexes is the protonated amino group on the side chain of the histamine moiety (hence histamineH is used in the formula) that gives compounds **1a **and** 1b** a neutral charge. This structural feature is however more common in NAMI-type compounds with a purine derivative as the nitrogen ligand [21]. The hydrogen atoms on the amino group (H3A, H3B and H3C) form hydrogen bonds with the four chloride atoms and the dmso oxygen. The Cl2 chloride atom forms an additional hydrogen bond with the hydrogen on the imidazole moiety (H2) which results in a slightly longer Ru-Cl2 distance (see Tables 2 and 3 and Figures 1 and 2). Compound **1a** exhibits an additional hydrogen bond between the water molecule and one of the chloride anions. It was not possible to locate the positions of two hydrogen atoms bonded to oxygen in a water molecule of the complex **1a** by difference Fourier maps.

**Figure 3:**
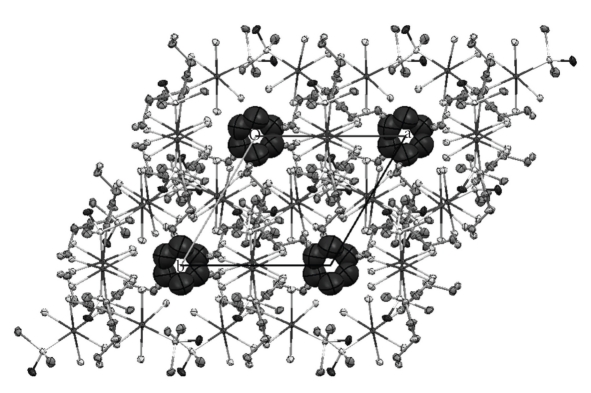
The packing in the crystal structure of compound **1a **along the *z *axis.

Another interesting feature of the crystal structure of compound **1a** is the formation of channels along *z* axis where the water molecules are located (see Figure 3).

### 3.2. Electrospray Ionization Mass Spectrometry

Compound **1b** was also characterized by electrospray ionization mass spectrometry. The peaks in the mass spectrum and their respective assignments are presented in Table 4. The data confirm a partial hydrolysis of the compound in water solution. Such behavior is usual for the NAMI-type compounds which undergo a rather quick dissociation of two of the chloride ligands and/or the dmso molecule [23].

### 3.3. Biological Activity

Previous studies have shown that while NAMI-A is inactive against B16F1 cells *in vitro*, it shows remarkable activity at relatively low concentrations when combined with electroporation [24]. On the other hand compound **1b** shows no activity at any of the tested concentrations (up to 1 mM) either by itself or in combination with electroporation. 

As Dyson and Sava suggest [25], the cytotoxicity of a compound as one of the main criteria in the preliminary screenings when looking for a potential drug candidate should be considered with some caution. NAMI-A for example, showed remarkable antimetastatic properties despite very low cytotoxicity. Further investigations of the biological activity of the histaminic analogue and the study of the interactions with different proteins are being planned.

## Supplementary Material

CCDC 759890 and 759818 contain the supplementary crystallographic data for **1a** and **1b**. These data can be obtained free of charge via http://www.ccdc.cam.ac.uk/conts/retrieving.html, or from the Cambridge Crystallographic Data Centre, 12 Union Road, Cambridge CB2 1EZ, UK; fax (+44) 1223-336-033; or e-mail: deposit@ccdc.cam.ac.uk.

## Figures and Tables

**Scheme 1:**
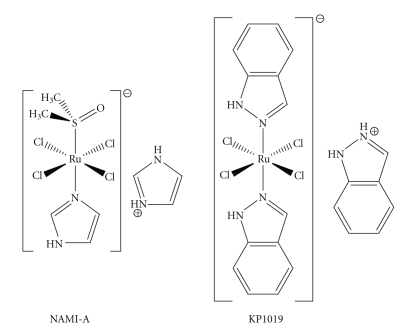
NAMI-A and KP1019.

**Table 1:** Crystal data and structure refinement for compounds **1a** and **1b**.

	Compound **1a**	Compound **1b**
Empirical formula	C_7_H_16_Cl_4_N_3_O_2_RuS	C_7_H_16_Cl_4_N_3_ORuS
Formula weight	449.16 g/mol	433.16 g/mol
Temperature	150(2) K	293(2) K
Wavelength	0.71073 Å	0.71073 Å
Crystal system	Triclinic	Monoclinic
Space group	*P 31 2 1*	*C* 2/*c *
Unit cell dimensions	*a* = 8.233 Å	*a* = 14.4860(8) Å
	*b* = 8.233 Å	*b* = 7.8741(3) Å
	*c* = 38.9269(3) Å	*c* = 27.3464(15) Å
	*α* = 90°	*α* = 90°
	*β* = 90°	*β* = 91.457(2)°
	*γ* = 120°	*γ* = 90°
Volume	2284.943(18) Å^3^	3118.2(3) Å^3^
*Z*	6	8
Density (calculated)	1.959 g/cm^3^	1.845 g/cm^3^
Absorption coefficient	1.864 mm^−1^	1.813 mm^−1^
*F*(000)	1338	1720
Crystal size	0.1 × 0.1 × 0.1 mm	0.08 × 0.05 × 0.05 mm
Theta range for data collection	3.26 to 28.71°	3.22 to 27.49°
Reflections collected	7263	5747
Independent reflections	3830 [*R*(int) = 0.0166]	3501 [*R*(int ) = 0.0290]
Refinement method	Full-matrix least-squares on *F* ^2^	Full-matrix least-squares on *F* ^2^
Data/restraints/parameters	3830/0/166	3501/0/172
Goodness-of-fit on *F * ^2^	1.068	1.080
Final *R* indices [*I * > 2 sigma(*I*)]	*R*_1_^a^ = 0.0236, *w* *R*_2_^b^ = 0.0571	*R*_1_ = 0.0500, *w* *R*_2_ = 0.1120
*R* indices (all data)	*R*_1_ = 0.0261, *w* *R*_2_ = 0.0583	*R*_1_ = 0.0761, *w* *R*_2_ = 0.1229
Largest diff. peak and hole	1.229 and −0.686 e·Å^3^	1.098 and −0.560 e·Å^−3^

^a^ *R*_1_ = ∑(|*F*_*o*_ | −|*F*_*c*_|)/∑|*F*_*o*_ |; ^b^ *w* *R*_2_ = {[*w*(*F*_*o*_^2^ − *F*_*c*_^2^)^2^]/∑[*w* *F*_*o*_^2^]}^1/2^.

**Table 2:** Selected bond lengths in compounds **1a**, **1b** and **NAMI** [22] (Na[RuCl_4_(*S*-dmso)(imidazole)]).

	**1a**	**1b**	**NAMI**
Ru–Cl_1_	2.355 (2)	2.359 (2)	2.3403 (9)
Ru–Cl_2_	2.364 (2)	2.364 (2)	2.3227 (8)
Ru–Cl_3_	2.356 (2)	2.343 (2)	2.3588 (9)
Ru–Cl_4_	2.358 (2)	2.356 (2)	2.3447 (8)
Ru–S	2.300 (2)	2.298 (2)	2.2956 (6)
Ru–N	2.096 (2)	2.097 (2)	2.081 (2)

**Table 3:** Selected hydrogen bond short contact distances in compound **1b**.

D–H ⋯ A	D–A (Å)	angle DHA (°)
N_2_–H_2_ ⋯ Cl_2_	3.218 (6)	151
N_3_–H_3_A ⋯ Cl_2_	3.331 (6)	138
N_3_–H_3_A ⋯ Cl_4_	3.306 (6)	140
N_3_–H_3_B ⋯ Cl_1_	3.308 (6)	149
N_3_–H_3_B ⋯ Cl_3_	3.349 (6)	131
N_3_–H_3_C ⋯ O_1_	2.889 (6)	172

**Table 4:** Main peaks in the ESI-MS spectrum of compound **1b** and respective assignments.

m/z	fragment
264	RuCl(histamine)(OH)
299	RuCl_2_(histamine)(OH)
325	RuCl(dmso)(histamine)
361	RuCl_2_(dmso)(histamine)
