# Supplementary material for: Synthesis and Characterization of Novel Ruthenium(III) Complexes with Histamine
Source: Bioinorg Chem Appl. 2010 Jun 2;2010:183097. doi: 10.1155/2010/183097 (PMC2901621; doi:10.1155/2010/183097)
Supplement: Supplementary file 1 — CCDC 759890 and 759818 contain the supplementary crystallographic data for 1a and 1b. These data can be obtained free of charge via http://www.ccdc.cam.ac.uk/conts/retrieving.html, or from the Cambridge Crystallographic Data Centre, 12 Union Road, Cambridge CB2 1EZ, UK; fax (+44) 1223-336-033; or e-mail: deposit@ccdc.cam.ac.uk. [file 183097.f1.pdf]

```
data_k825

_publ_contact_author_name        'Jakob Kljun'
_publ_contact_author_address


;
             Faculty of Chemistry and Chemical Technology
             University of Ljubljana
             A\<sker\<ceva 5, P. O. Box 537
             1000 Ljubljana
             Slovenia
;
_publ_contact_author_phone       '+386 1 2419 100'

_publ_contact_author_fax         '+386 1 2419 220'

_publ_contact_author_email       'jakob.kljun@fkkt.uni-lj.si'

_publ_requested_journal 'Bioinorganic Chemistry and Applications'

_publ_author_name                'Kljun, J.','Petri\<cek, S.','\<Zigon, D.','Hudej, R.','Miklav\<ci\<c,
D.','Turel, I.'

_audit_creation_method  SHELXL-97
_chemical_name_systematic
;
 trans tetrachlorido-(dimethylsulfoxide)-(4-(2-ammonioethyl)imidazole) ruthenium(III) monohydrate
;
_chemical_name_common            ?
_chemical_melting_point ?
_chemical_formula_moiety         ?
_chemical_formula_sum
 'C7 H18 Cl4 N3 O2 Ru S'
_chemical_formula_weight         451.16

loop_
 _atom_type_symbol
 _atom_type_description
 _atom_type_scat_dispersion_real
 _atom_type_scat_dispersion_imag
 _atom_type_scat_source
 'C'  'C'   0.0033   0.0016
 'International Tables Vol C Tables 4.2.6.8 and 6.1.1.4'
 'H'  'H'   0.0000   0.0000
 'International Tables Vol C Tables 4.2.6.8 and 6.1.1.4'
 'N'  'N'   0.0061   0.0033
 'International Tables Vol C Tables 4.2.6.8 and 6.1.1.4'
 'O'  'O'   0.0106   0.0060
```
