# Supplementary material for: Synthesis and Characterization of Novel Ruthenium(III) Complexes with Histamine
Source: Bioinorg Chem Appl. 2010 Jun 2;2010:183097. doi: 10.1155/2010/183097 (PMC2901621; doi:10.1155/2010/183097)
Supplement: Supplementary file 2 [file 183097.f2.pdf]

```
data_k1684

_publ_contact_author_name        'Jakob Kljun'
_publ_contact_author_address


;
            Faculty of Chemistry and Chemical Technology
            University of Ljubljana
            A\<sker\<ceva 5, P. O. Box 537
            1000 Ljubljana
            Slovenia
;
_publ_contact_author_phone       '+386 1 2419 100'

_publ_contact_author_fax         '+386 1 2419 220'

_publ_contact_author_email       'jakob.kljun@fkkt.uni-lj.si'

_publ_requested_journal 'Bioinorganic Chemistry and Applications'

_publ_author_name                'Kljun, J.','Petri\<cek, S.','\<Zigon, D.','Hudej, R.','Miklav\<ci\<c,
D.','Turel, I.'

_audit_creation_method  SHELXL-97
_chemical_name_systematic
;
 trans tetrachlorido-(dimethylsulfoxide)-(4-(2-ammonioethyl)imidazole) ruthenium(III)
;
_chemical_name_common            ?
_chemical_melting_point ?
_chemical_formula_moiety         'C7 H16 Cl4 N3 O Ru S'
_chemical_formula_sum
 'C7 H16 Cl4 N3 O Ru S'
_chemical_formula_weight         433.16

loop_
 _atom_type_symbol
 _atom_type_description
 _atom_type_scat_dispersion_real
 _atom_type_scat_dispersion_imag
 _atom_type_scat_source
 'C'  'C'    0.0033    0.0016
 'International Tables Vol C Tables 4.2.6.8 and 6.1.1.4'
 'H'  'H'    0.0000    0.0000
 'International Tables Vol C Tables 4.2.6.8 and 6.1.1.4'
 'N'  'N'    0.0061    0.0033
 'International Tables Vol C Tables 4.2.6.8 and 6.1.1.4'
 'O'  'O'    0.0106    0.0060
```
